# Carbon Ion irradiation in the treatment of grossly incomplete or unresectable malignant peripheral nerve sheaths tumors: acute toxicity and preliminary outcome

**DOI:** 10.1186/s13014-015-0414-8

**Published:** 2015-05-06

**Authors:** Alexandra D Jensen, Matthias Uhl, Naved Chaudhri, Klaus K Herfarth, Juergen Debus, Falk Roeder

**Affiliations:** Department of Radiation Oncology, University of Heidelberg, INF 400, 69120 Heidelberg, Germany; Department of Medical Physics, Heidelberg Ion Beam Therapy Centre (HIT), Heidelberg, Germany; Department of Molecular and Radiation Oncology, German Cancer Research Center (DKFZ), Heidelberg, Germany; Clinical Cooperation Unit Radiation Oncology, German Cancer Research Center (DKFZ), Heidelberg, Germany

## Abstract

**Background:**

To report our early experience with carbon ion irradiation in the treatment of gross residual or unresectable malignant peripheral nerve sheath tumors (MPNST).

**Methods:**

We retrospectively analysed 11 patients (pts) with MPNST, who have been treated with carbon ion irradiation (C12) at our institution between 2010 and 2013. All pts had measurable gross disease at the initiation of radiation treatment. Median age was 47 years (29-79). Tumors were mainly located in the pelvic/sacral (5 pts) and sinunasal/orbital region (5 pts). 5 pts presented already in recurrent situation, 3 pts had been previously irradiated, and in 3 pts MPNST were neurofibromatosis type 1 (NF1) associated. Median cumulative dose was 60 GyE. Treatment was carried out either as a combination of IMRT plus C12 boost (4 pts) or C12 only (7 pts).

**Results:**

Median follow-up was 17 months (3-31 months). We observed 3 local progressions, translating into estimated 1- and 2-year local control rates of 65%. One patient developed distant failure, resulting in estimated 1- and 2-year PFS rates of 56%. Two patients have died, therefore the estimated 1- and 2-year OS rates are 75%. Acute radiation related toxicities were generally mild, no grade 3 side effects were observed. Severe late toxicity (grade 3) was scored in 2 patients (trismus, wound healing delays).

**Conclusion:**

Carbon ion irradiation yields very promising short term local control and overall survival rates with low morbidity in patients suffering from gross residual or unresectable malignant peripheral nerve sheath tumors and should be further investigated in a prospective trial.

## Background

Malignant peripheral nerve sheath tumors (MPNSTs) comprise a rare group of tumors that arise from different cells found in the sheath of peripheral nerves including Schwann cells, perineural fibroblasts or fibroblasts, formerly known as malignant schwannoma, neurofibrosarcoma, neurogenic sarcoma or malignant neurilemoma [1]. The reported incidence is 1:1000000 per year [2], representing 5-10% of all malignant soft tissue tumors [3]. The most important risk factor is neurofibromatosis type I (NF I), which accounts for about half of the cases [1]. The incidence seems equal in males and females and the most common age at diagnosis is 20 to 50 years [2]. Predominant sites of presentation are the proximal extremities and the pelvis [4]. They usually present as enlarging mass, which may be associated with pain or neurological symptoms [2]. However, MPNSTs are highly aggressive tumors characterized by rapid, infiltrating growth and hematogenous dissemination [1]. The preferred modality for imaging of MPNSTs is MRI [5] for the primary lesion and CT chest to exclude pulmonary metastases [6].

Complete resection with negative margins remains the mainstay of treatment but frequently results in functional deficits due to the inherent necessity to sacrifice the involved peripheral nerves [7]. Even after microscopic complete resection, local recurrence rates still approach 20-38% [3,4,8], which is even increasing in the presence of unfavourable prognostic factors such as positive margins/gross residual disease, recurrent situation at presentation or head and neck as initial presentation site [4]. Therefore, neoadjuvant or adjuvant radiation is commonly used as generally recommended in high grade soft tissue sarcomas [2,9]. If complete surgical resection seems impossible due to vital adjacent structures or would result in inacceptable functional deficits, primary definitive radiation therapy can be an alternative in selected cases [10]. Due to the relatively low radiation sensitivity of these tumors, doses approaching 70 Gy are needed in conventional fractionated photon therapy in order to achieve long-term local control in the presence of gross residual disease [10]. Application of these doses would result in considerable acute and late toxicity, and therefore cannot be achieved very often [10]. Especially in cases with directly adjacent radiosensitive structures, charged particles such as protons or carbon ions can offer superior dose distributions even compared to sophisticated photon techniques [11]. Furthermore, carbon ions seem to have a higher relative biological effectiveness (RBE) compared to photons or protons, which may result in increased efficacy as shown in other radioresistant diseases [11]. For these reasons, patients with unresectable or incompletely resected MPNSTs were offered carbon ion treatment at our institution. Here we report our first experience.

## Methods

The medical records of 11 consecutive patients with MPNST treated with carbon ion radiation in our institution between 2010 and 2013 were reviewed. Median age was 47 years (range 29 – 79 years) and 6 patients were male. Tumors were located in the pelvic/sacral (5), sinunasal/orbital (5) and cervical region (1). Disease was histologically confirmed in all patients. Eight patients had been surgically treated by partial resection, while 3 patients received biopsy only. Measurable gross disease was present in all patients prior to radiation therapy. Two patients had received induction chemotherapy with adriamycin/ifosfamide, but no systemic therapy was applied concurrently to radiation. Five patients presented already in recurrent situation, 3 of whom had been previously irradiated. All except one patient showed high grade lesions. MPNST was neurofibromatosis type 1 (NF1) associated in 3 patients.

Initial work-up included at least clinical examination, MRI of the primary lesion and CT chest. Patients were immobilized with a thermoplastic head mask or a vacuum pillow in supine position. Treatment planning was based on contrast-enhanced CT (3 mm slice thickness) in correlation with MRI. The first three patients were treated with combination of photons (IMRT or Tomotherapy) and carbon ions, while the following 8 patients were treated with carbon ions only. The GTV included all visible gross disease. The CTV for the carbon ion treatment included the GTV with a safety margin of 3 to 5 mm. In cases with involvement of paranasal sinus all surgically targeted sinus were included into the CTV. Another 3 mm were added to generate the PTV. Margins could be reduced at anatomical borders or to spare directly adjacent radiosensitive organs (i.e. spinal cord, optic system) at risk at the discretion of the treating physician. In combination treatments, a second PTV (PTV2) was generated with a margin of 5 cm along typical pathways of spread. In the head and neck, the PTV2 included typical compartments, but could not be extended to 5 cm due to proximity of critical structures. Treatment planning for carbon ion therapy was performed using TPS© Siemens planning software for inversely planned, intensity-controlled carbon ion therapy in raster-scanning technique [12]. Biological effectiveness of the particle beam was incorporated in the planning software baseline according to the Local Effects Model (LEM) [13]. The total dose was prescribed to the median of the PTV and yielded photon Gray equivalent (GyE). No further calculations were needed to take account of the increased biological effectiveness of carbon ion beams. Note that variations in the biological effectiveness due to changes in fractionation size are not included in this model, doses need to be converted according to the standard LQ model. Treatment planning aims for coverage of the CTV by the 95% and the PTV by the 90% isodose according to ICRU 83 [14] . Carbon ion therapy was clinically available at the HIT facility for 5-6 days per week.

**Figure 1:**
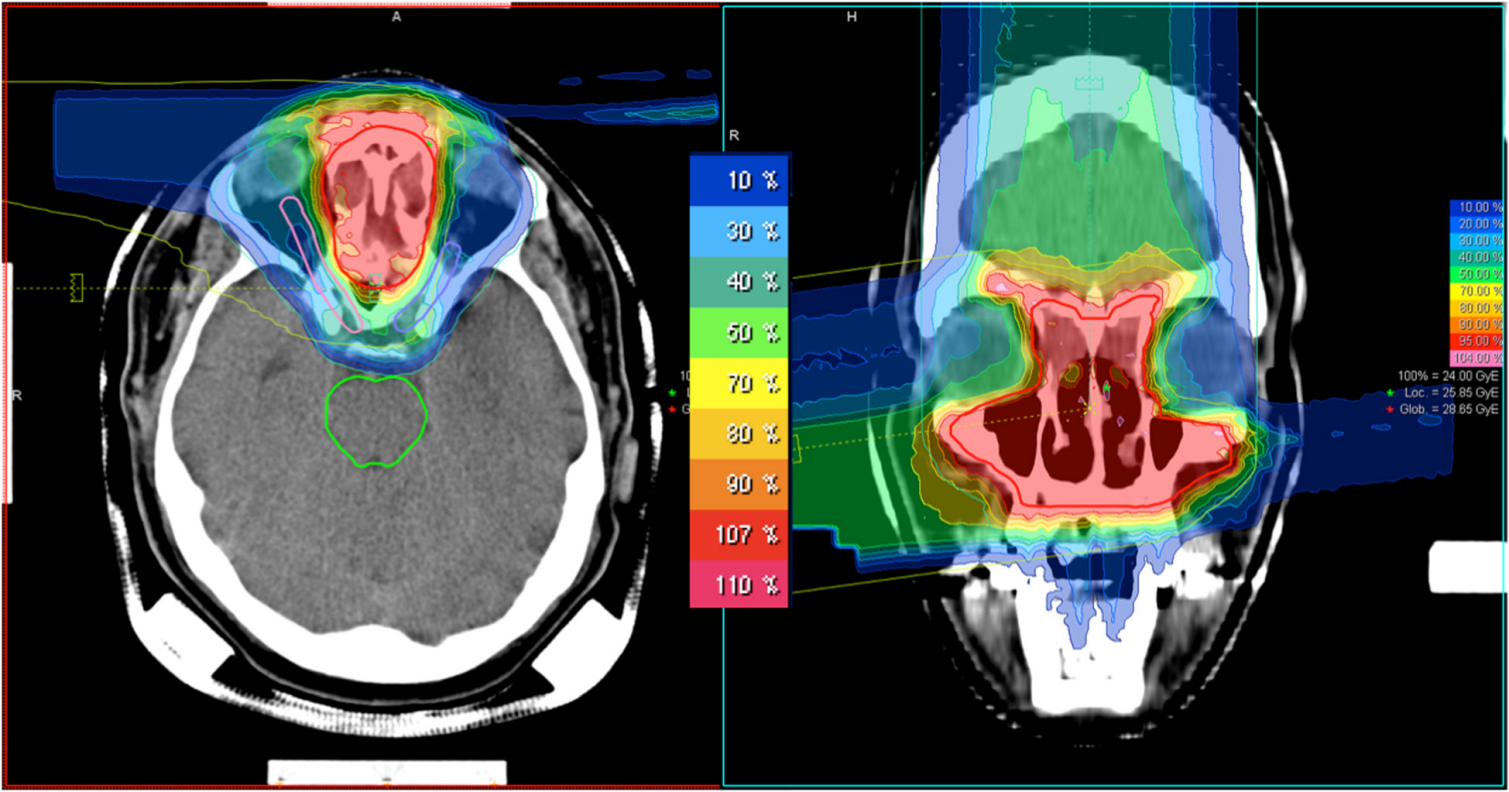
Example for a dose distribution in a patient with paranasal MPNST (3-field IMPT).

**Figure 2:**
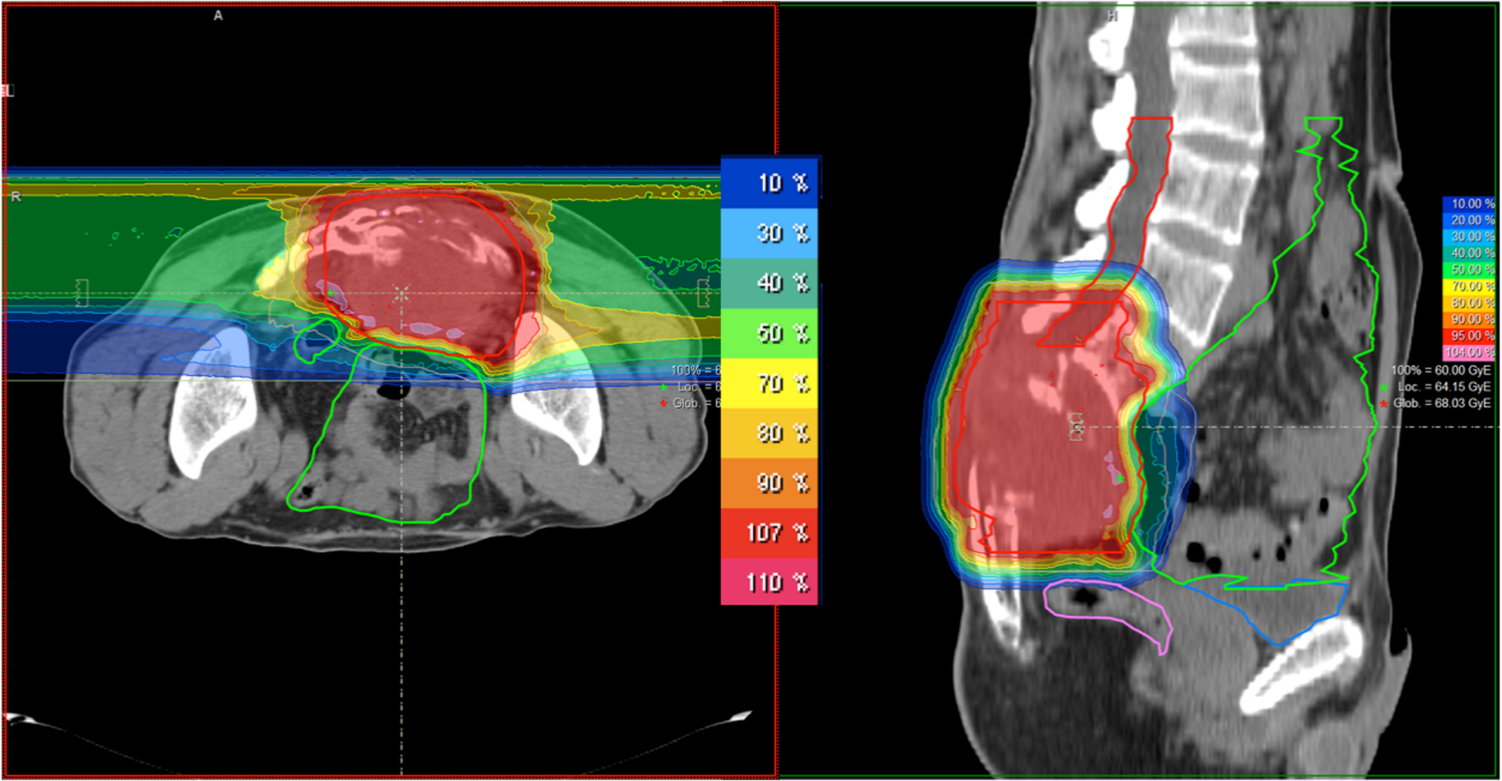
Example for a dose distribution in a patient with sacral MPNST (2-field IMPT).

All patients with combined treatment received 50 Gy photon IMRT in 25 fractions and 24 GyE carbon ions in 8 fractions. Total cumulative dose in these cases is 74 GyE corresponding to 80 Gy BED. Patients receiving carbon ions only were treated to total doses of 60 - 66 GyE in 20-22 fractions (median 60 GyE). Patients who have had prior radiation therapy were re-irradiated with 54-60 GyE in 18-20 fractions. Carbon ion radiation was applied in intensity-controlled raster-scanning technique (active beam application) using one to three treatment angles (median: 2) as described in detail earlier [15]. Examples of three dimensional dose distributions for patients with tumors located in the paranasal sinus and sacral region are shown in Figure 1 and 2.

Regular follow up took place at our institution or the referring center including at least clinical examination with scoring of toxicity and MRI of the primary lesion every 3 months for the first two years and every 6 months thereafter. CT chest was performed at least every second visit. In case of clinical evidence of local recurrence or distant spread, additional tests or imaging modalities were performed to confirm or exclude disease progression at the discretion of the treating physician. Acute and late toxicity were scored according to CTCAE v4.0. Time to event data was calculated from the first day of irradiation until the last follow up information or until death using the Kaplan-Meier method. Locoregional control was defined as the absence of tumor regrowth in the region of the treated lesion on repeated MRI scans based on best response after treatment. Progression-free survival was defined as absence of local or distant progression or death of any cause. No subgroup analyses were performed due to the small sample size. This work is in accordance with the Declaration of Helsinki in its most recent version. Retrospective analysis was approved by the University of Heidelberg ethics committee (S-141/2014). All patients gave written informed consent prior to initiation of treatment.

## Results

**Figure 3:**
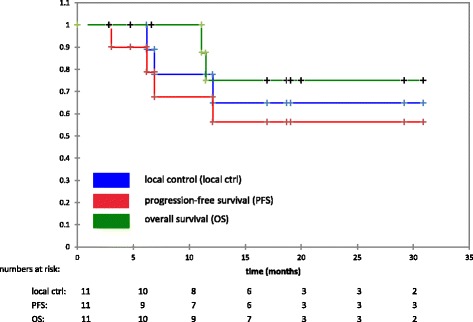
Local control, progression-free survival and overall survival (entire cohort).

The median follow-up for the entire cohort was 17 months (3-31) and 19 months in surviving patients. We observed 3 local progressions so far, translating into estimated 1- and 2-year local control rates of 65% (see Figure 3). One of the recurrences was clearly in-field while 2 were located at the borders of the high-dose area. Two out of five patients (40%) in recurrent situation and one out of 6 patients (17%) in primary situation developed local progressions. One patient with local progression had been previously irradiated. One additional patient developed distant failure. Overall, 4 patients showed local or distant disease progression. The estimated 1- and 2-year progression-free survival rates were 56% (see Figure 3). Two patients have deceased so far, both disease-related. The resulting estimated 1- and 2-year overall survival rates were 75% (see Figure 3).

Maximum acute radiation related side effects were grade 1 in five patients and grade 2 in one patient, mainly dermatitis and xerophthalmia. No grade 3 acute radiation related toxicities were observed. For detailed acute toxicity see Table 1. Severe late toxicities (grade 3) were scored in 2 patients, one suffered from trismus after primary treatment of a large lesion located in the sphenoid sinus with involvement of the cavernous sinus (and one from visual impairment). The other patient had undergone partial resection of a large sacral MPNST. In view of the tumor’s aggressive growth pattern and prior radiotherapy, it was decided to start radiotherapy as early as possible following interdisciplinary discussion. Unfortunately but not unexpectedly, this patient developed wound healing delays after re-irradiation of the sacral region. For detailed late toxicity see Table 2.

**Table 1:** **Acute radiation related toxicity**

**Acute toxicity**	**°I**	**°II**
**(CTCAE v.4)**	**(x/11 pts)**	**(x/11 pts)**
Dermatitis	4	1
Dysphagia	1	
Dysgeusia	2	
Xerophthalmia	3	
Conjunctivitis	1	
Trigeminus neuralgia	1

**Table 2:** **Late radiation related toxicity**

**Late toxicity (CTCAE v.4)**	**x/11 pts**
Dermatitis °I	1 pt
Hyperpigmentation °I	1 pt
Visual impairment	1 pt
Delayed wound healing	1 pt
Trismus	1 pt

## Discussion

Here we report a retrospective case series of irresectable or incompletely resected malignant peripheral nerve sheath tumors treated with carbon ion radiation therapy. To our knowledge, our work represents the largest series ever published in peer-reviewed literature focusing especially on the use of carbon ion therapy in this disease. With a median follow-up of 17 months, we observed a crude incidence of local and distant failures of 27% and 9%, respectively, translating into 2-year local control, progression-free survival and overall survival rates of 65%, 56% and 75%, respectively. These results seem to be promising given the very unfavourable prognostic factors in our patient cohort.

The cornerstone of curative intent treatment of MPNST usually remains complete excision with wide margins [2]. Even after complete excision, local recurrence rates of 20-38% have been reported in large series, prompting many authors to recommend additional radiation therapy [3,4,8]. Non-extremity site, recurrent situation at presentation, and positive margins have been consistently identified as factors significantly associated with reduced local control and survival in many series [3,4,7,8,16,17]. Already the presence of microscopically positive margins was strongly associated with significantly increased local recurrence rates, ranging from 61% to 100 % [3,4,8]. Data on gross residual disease in resected patients are rare, but outcome was usually very poor in the small numbers of cases published. For example, Minovi et al. [1] reported on 3 cases in their series of 17 patients with incomplete resections and gross residual disease. Two of them developed local recurrences shortly after surgery and all of them finally died of disease. Sordillo et al. [16] observed only 10 patients in their series of 165 patients with non-surgical-treatment, none of them was free of disease at 3 years. Hruban et al. [18] reported 2 out of 43 patients with subtotal resections, both developed local recurrences and died of disease. Wanebo et al. [17] included 9 patients with gross residual disease in their series of 28 patients. Median progression-free survival for those was only 1 month with a 1-year PFS rate of less than 25% and median overall survival was about 9 months with 1- and 2-year rates of roughly 40% as estimated from the survival curves. Ducatman et al. [7] reported on 46 patients with gross residual disease after surgery, of whom the majority received additional radiation. These tumors were mainly located in the head and neck or the retroperitoneal/pelvic area, similarly to our cases. The 1-year and 2-year OS survival rates were 63% and 43% as estimated from survival curves for these patients with no information given about local control. Given the very limited data specifically addressing the outcome of MPNST with gross residual tumor following resection, larger series including other types of soft tissue sarcoma treated with photon radiation therapy might serve as a benchmark. Slater et al. [19] analysed 72 patients treated with photon irradiation and found 2- and 5-year LC rates of 39% and 29%, respectively. Tepper et al. [20] described a similar 5-year LC rate of 33% in their 51 patients. Kepka et al. [10] reported a very large series of 112 patients with unresectable sarcomas treated with photon radiation and found 2-year local control, disease-free survival and overall survival rates of 52%, 33% and 55%, respectively. In our cohort, all patients suffered from non-extremity tumors, 45% were in recurrent situation and all had gross residual disease after surgery. Although comparisons between different retrospective series are always difficult and potentially flawed with biases, our results seem to compare favourably with the published series of patients with grossly incomplete resections of MPNST or soft tissue sarcomas, especially given the presence of unfavourable prognostic factors.

As soft tissue sarcomas including MPNST are generally thought to be comparatively radioresistant tumors, high doses are usually recommended even after gross complete resections. For example Wong et al. [3] analysed 73 patients with MPNST who received adjuvant radiation and described a significantly improved 5-year local control rate of 73% with doses of 60 Gy or more compared to 50% if doses <60 Gy were applied. This dose dependency was confirmed by multivariate analysis. Similar dose effect relationships have been described for soft tissue sarcomas after gross total resection. For example Wolfson et al. suggested [21] that doses above 63 Gy favourably affect survival. Zagars et al. [22] found improved local control rates for doses between 64 and 68 Gy compared to 60 Gy and Fein et al. [23] demonstrated improved local control rates if doses of 65 Gy or more were used. For gross residual disease, even higher doses have to be attempted to achieve durable local control at least in a substantial proportion of patients. For example, Carli et al. [24] recommended doses of 65-70 Gy for gross residual disease based on their findings in pediatric MPNST. Tepper et al. [20] found a significantly improved local control rate with doses of more than 64 Gy in a series of unresectable soft tissue sarcoma. Slater et al. [19] described longer duration of local control after doses exceeding 65 Gy and Kepka et al. [10] reported significantly improved local control, disease-free survival and overall survival rates in unresectable soft tissue sarcoma patients treated with doses of 63 Gy or more. They confirmed their results in a multivariate analysis and calculated an improvement of 3% per Gy in the 5-year local control and overall survival rate. However, possible improvements in local control by dose-escalation have always to be weighed against toxicity and functional outcome. For example Mundt et al. [25] observed a severe complication rate of 0% for doses < 63 Gy compared to 23% with doses exceeding 63 Gy in grossly resected soft tissue sarcomas. Stinson et al. [26] also described significantly worse functional outcomes for doses of more than 63 Gy. Kepka et al. [10] described a major complication rate of 8% for doses less or equal to 68 Gy compared to 27% for doses exceeding 68 Gy in unresectable soft tissue sarcomas, and Slater et al. [19] observed 5 of 6 severe complications in patients who were treated with 70 Gy or more. This small therapeutic window prompted several groups including ours to investigate alternative boosting techniques like intraoperative radiation therapy (IORT) or brachytherapy to overcome the limitations of external beam radiation therapy, especially in non-extremity regions [3,9,27-32]. For example, Wong et al. [3] observed significantly improved local control rates according to univariate and multivariate analysis of their series of grossly resected MPNSTs, if IORT or brachytherapy was used as a boosting technique compared to sole percutaneous adjuvant radiation therapy. Oertel et al. [33] showed a low toxicity profile for the combination of IORT with moderate doses of EBRT while achieving similar 5-year local control rates after gross incomplete resection of soft tissue sarcomas as reported with high dose external beam radiation. However, experience in dose-escalated high-precision photon techniques is limited: to our knowledge, no data has been presented on the effect of photon doses exceeding 70 Gy.

Another possibility to enhance the radiation effect could be the use of different radiation types like particles mainly because of their known higher biological effectiveness compared to photon irradiation. Therefore several groups introduced fast neutrons into the treatment of soft tissue sarcomas, which resulted in promising local control rates for gross disease but was accompanied by considerable late toxicities [34,35]. In contrast to neutrons, charged particles like protons and especially carbon ions offer not only an increased biological effectiveness, but also very conformal dose distributions applying lower doses to adjacent organs at risk compared to even the most sophisticated photon techniques as shown by various dosimetric studies [36]. The superior dose distribution is mainly based on a unique phenomenon of charged particles known as the Bragg peak. In contrast to photons, charged particles enter and travel through tissue with minimal dose deposition along their path until a peak of deposition is reached in an energy-dependent depth. Beyond the so called Bragg peak, the dose deposition practically falls to zero. These steep gradients allow for superior sparing of directly adjacent organs at risk [36,37]. In contrast to photons or protons, carbon ions are additionally characterized by a high linear energy transfer resulting in different radiobiological interactions with tumor and normal tissue with an enhanced relative biological effectiveness. Therefore carbon ions seem to be a very attractive treatment option especially for unresectable or incompletely resected tumors with known radioresistant histology located directly adjacent to radiosensitive organs at risk. The transfer of these theoretical advantages into improved local control rates while consistently maintaining a very low toxicity profile has already been shown in several diseases fulfilling above mentioned criteria, for example chordoma or chondrosarcoma of skull base or sacral region [11,15,38]. In our cohort, we also observed very promising local control rates while the rate of severe toxicities was considerably low, especially if taken into account, that our median dose of 60 GyE would be equivalent to about 74 Gy of conventionally fractionated photon radiation as calculated with the currently available models.

Clearly our study has some limitations, namely its retrospective nature, the small sample size and the short follow up. Nevertheless it shows very promising results in an unfavourable patient group and therefore adds valuable information to the existing literature.

## Conclusion

Carbon ion irradiation yielded very promising short term local control and overall survival rates with low morbidity in patients suffering from grossly incomplete or unresectable malignant peripheral nerve sheath tumors and should be further investigated in a prospective trial.

## Abbreviations

BED: Biological effective dose
CT: Computed tomography
CTCAE: Common Terminology Criteria for Adverse Events
CTV: Clinical target volume
EBRT: External beam radiotherapy
GTV: Gross tumor volume
Gy: Gray
GyE: Gray equivalent
ICRU: The international commission on radiation units and measurements
IORT: Intraoperative radiation therapy
LC: Locoregional control
LEM: Local effects model
LQ: Linear quadratic
MPNST: Malignant peripheral nerve sheath tumors
MRI: Magnetic resonance imaging
NF: Neurofibromatosis
OS: Overall survival
PFS: Progression-free survival
PTV: Planning target volume
RBE: Relative biological effectiveness
